# Prevalence, incidence and clinical outcomes of epicardial coronary artery disease among transthyretin amyloidosis cardiomyopathy patients

**DOI:** 10.1186/s12872-023-03140-y

**Published:** 2023-03-08

**Authors:** Rana Hassan, Robert J. H. Miller, Jonathan G. Howlett, James A. White, Nowell M. Fine

**Affiliations:** Division of Cardiology, Department of Cardiac Sciences, Alberta Health Services, Libin Cardiovascular Institute, Cumming School of Medicine, University of Calgary, South Health Campus, 4448 Front Street SE, Calgary Alberta, T3M 1M4 Canada

**Keywords:** Transthyretin amyloidosis cardiomyopathy, Coronary artery disease, Prevalence, Mortality, Amyloidosis

## Abstract

**Background:**

Transthyretin amyloidosis cardiomyopathy (ATTR-CM) patients are often older and may be at risk for obstructive epicardial coronary artery disease (oeCAD). While ATTR-CM may cause small vessel coronary disease, the prevalence and clinical significance of oeCAD is not well described.

**Methods and results:**

The prevalence and incidence of oeCAD and its association with all-cause mortality and hospitalization among 133 ATTR-CM patients with ≥ 1-year follow-up was evaluated. The mean age was 78 ± 9 years, 119 (89%) were male, 116 (87%) had wild-type and 17 (13%) had hereditary subtypes. Seventy-two (54%) patients underwent oeCAD investigations, with 30 (42%) receiving a positive diagnosis. Among patients with a positive oeCAD diagnosis, 23 (77%) were diagnosed prior to ATTR-CM diagnosis, 6 (20%) at the time of ATTR-CM diagnosis, and 1 (3%) after ATTR-CM diagnosis. Baseline characteristics between patients with and without oeCAD were similar. Among patients with oeCAD, only 2 (7%) required additional investigations, intervention or hospitalization after ATTR-CM diagnosis. After a median follow-up of 27 months there were 37 (28%) deaths in the study population, including 5 patients with oeCAD (17%). Fifty-six (42%) patients in the study population required hospitalization, including 10 patients with oeCAD (33%). There was no significant difference in the rates of death or hospitalization among ATTR-CM patients with and without oeCAD, and oeCAD was not significantly associated with either outcome by univariable regression analysis.

**Conclusions:**

While oeCAD is prevalent in ATTR-CM patients, this diagnosis is frequently known at time of ATTR-CM diagnosis and characteristics are similar to patients without oeCAD.

## Introduction

Transthyretin amyloidosis cardiomyopathy (ATTR-CM) is an important and underrecognized cause of heart failure (HF) [ 1]. Both ATTR-CM subtypes, the hereditary type (hATTR) caused by a transthyretin (TTR) gene mutation, and especially the wild-type (wtATTR), an age-related disorder occurring in the absence of a TTR gene mutation, often occur in older patients [2]. As a result, many ATTR-CM patients have a significant burden of age-related comorbid conditions, including cardiovascular comorbidities. Patients with ATTR-CM can develop biventricular heart failure (HF) that progresses to a restrictive cardiomyopathy phenotype with variable systolic function [1]. Other recognized cardiovascular manifestations may include atrial arrhythmias, conduction system disease, aortic valve stenosis, and autonomic dysfunction [1].

Cardiac amyloidosis patients may present with symptoms of ischemic heart disease. This has been frequently attributed to intramural microvascular amyloid fibril deposition leading to microvascular ischemia, which has been predominantly associated with the light chain (AL) amyloidosis subtype [3–5]. While histopathologic studies have identified the presence of amyloid deposits in epicardial coronary artery walls, this does not commonly lead to obstructive disease resulting in a clinical diagnosis of coronary artery disease [4, 6]. Given their frequently older age, ATTR-CM patients may have a meaningful prevalence of concurrent atherosclerotic obstructive epicardial coronary artery disease (oeCAD). Furthermore, it has been speculated that epicardial coronary artery amyloid deposits may accelerate existing atherosclerotic epicardial coronary disease [7, 8]. The prevalence and incidence of a clinical diagnosis of oeCAD in patients with ATTR-CM, along with its prognostic significance, is not well described. This may have direct relevance for the management of ATTR-CM patients because some medications frequently used for the treatment of oeCAD, such as beta-blockers and afterload reducing agents, are often poorly tolerated [1]. Approval of the TTR-stabilizing agent tafamidis may improve the prognosis for ATTR-CM patients, increasing the relevance of concurrent oeCAD in this population [9]. The purpose of this analysis was to describe the prevalence of clinically diagnosed oeCAD among ATTR-CM patients and its respective association with adverse clinical outcomes.

## Methods

### Study population

Consecutive patients followed at the Cardiac Amyloidosis Clinic at our institution with a diagnosis of ATTR-CM from November 2016 to January 2021 were included in this retrospective cohort analysis. Patients with both ATTR subtypes were included if they met the following criteria; (1) exclusion of light-chain (AL) amyloidosis through the absence of serum and urine monoclonal protein, (2) evidence of cardiac amyloidosis by either myocardial biopsy or positive technetium-99 m-pyrophosphate nuclear scintigraphy defined by grade 2–3 myocardial uptake or heart-to-contralateral lung ratio > 1.5, as previously described [10], and (3) either hATTR or wtATTR based upon results of genetic testing or proteomic analysis by mass spectrometry performed on biopsy tissue samples. Patients with non-ATTR subtypes of amyloidosis or those with < 12 months clinical follow-up were excluded. Clinical, medication, biochemical and cardiac imaging data were collected at the time of ATTR-CM diagnosis. This study was approved by the University of Calgary Research Ethics Board, and the requirement for informed written patient consent was waived.

### Coronary artery disease

All patients included underwent review of medical records for evidence of oeCAD by two study investigators (R.H., N.M.F.), including symptom history, cardiovascular risk factors, healthcare encounters such as ambulatory clinic visits, Emergency Department visits and hospitalizations, and cardiac investigation findings such as electrocardiogram (ECG), cardiac biomarker (troponin and N-terminal pro-B-type natriuretic peptide, NTproBNP), ECG stress test, stress imaging, coronary computed tomography angiography (CCTA), invasive coronary angiography, history of acute coronary syndrome (ACS) or myocardial infarction (MI), and/or coronary artery revascularization. The clinical indication for oeCAD evaluation, in addition to the temporal relation with ATTR-CM diagnosis (occurring before, after, or simultaneous with) was also collected.

As patients with ATTR-CM often have clinical characteristics and/or non-invasive investigation result findings that resemble oeCAD (such as chest pain, chronically elevated troponin levels, and anterior Q-waves on ECG), a strict definition of oeCAD was used for this analysis. A diagnosis of CAD required ≥ 1 of the following criteria: (1) prior history of coronary artery revascularization by either percutaneous coronary intervention (PCI) and/or coronary artery bypass grafting (CABG), (2) obstructive epicardial coronary artery stenosis of ≥ 70% by CCTA or invasive coronary angiography, or ≥ 50% of the left main coronary artery [11]. This strict criteria was selected in order to definitively confirm the presence of obstructive epicardial coronary artery disease lesions in ATTR-CM patients, and to discriminate the presence oeCAD from patients who may have microvascular coronary artery disease or findings on non-invasive evaluation (such as ECG or echocardiography) that are secondary to myocardial amyloid fibril infiltration but resemble oeCAD. Among patients with a prior history of ACS/MI, all had subsequent confirmatory invasive coronary angiography.

### Statistical analysis

Categorical variables are reported as frequency (percentage) and compared with a Chi-square or Fisher exact test. Continuous variables are reported as mean (standard deviation, SD) and compared with the student’s t-test if normally distributed or as the median (interquartile range, IQR) and compared with the Wilcoxon rank sum test if non-normally distributed, as determined using the Shapiro–Wilk test. Associations between the presence of oeCAD and clinical, cardiovascular risk factor, ATTR-related, medication, biochemical and imaging characteristics were examined using logistic regression analysis. The primary outcome was all-cause mortality, and the secondary outcome was all-cause hospitalization, further categorized as cardiovascular and non-cardiovascular hospitalization. Univariable Cox proportional hazards regression modeling was used to identify whether the presence of oeCAD or the presence of cardiovascular risk factors (hypertension, diabetes mellitus, hyperlipidemia or smoking history) was associated with study outcomes all-cause death and a composite of all-cause death or all-cause hospitalization, with hazard ratios (HR) and 95% confidence intervals (CIs) reported. Due to the limited sample size and number of events occurring in this cohort, multivariable Cox proportional hazards regression modelling was not performed. All probability values were 2-sided, and a value of ≤ 0.05 was considered statistically significant. Statistical analyses were performed using Stata software version 17.0 (College Station, Texas).

## Results

### Study population

Out of 162 eligible ATTR-CM patients, 11 were excluded due to insufficient available data in their medical records to exclude or confirm a diagnosis of oeCAD, and 18 were excluded for < 12 months follow-up, for a final study population of 133. Baseline characteristics of the study population and for those with wtATTR versus hATTR subtypes are shown in Table 1. The majority of ATTR-CM patients were male and had wtATTR. Among hATTR patients, the most common TTR gene mutations were pV142I, pT80A and pV30M. Other mutations represented in the cohort include one patient each with a pI127V, pI104T and pI88L mutation. wtATTR patients were significantly older, had a higher NYHA functional class and a higher proportion taking diuretics, while a significantly higher proportion of hATTR patients were taking an ATTR disease modifying agent (Table 1).

**Table 1:** Baseline characteristics of the study population and for patients with wtATTR and hATTR

Parameter	Total (*N* = 133)	wtATTR (*n* = 116)	hATTR (*n* = 17)	*p*-value
Clinical Characteristics				
Age (years)	78 ± 9	80 ± 8	66 ± 8	< 0.01
Male	119 (89%)	106 (91%)	13 (76%)	0.08
Body mass index (kg/m^2^)	27 ± 4	26 ± 5	27 ± 5	0.94
NYHA functional class III-IV	72 (54%)	67 (58%)	5 (29%)	0.04
Atrial fibrillation	48 (36%)	44 (38%)	4 (24%)	0.29
Hypertension	47 (35%)	35 (30%)	12 (71%)	< 0.01
Diabetes Mellitus	24 (18%)	23 (20%)	1 (6%)	0.31
Hyperlipidemia	36 (27%)	34 (29%)	2 (12%)	0.15
Smoking history	17 (13%)	14 (12%)	3 (18%)	0.46
hATTR mutation				
pV142I	5 (4%)	–	5 (29%)	–
pV50M	5 (4%)	–	5 (29%)	–
pT80A	4 (3%)	–	4 (24%)	–
Other	3 (2%)	–	3 (18%)	–
Medications				
Diuretics	90 (68%)	84 (72%)	6 (35%)	< 0.01
Anticoagulation	53 (40%)	49 (42%)	4 (24%)	0.19
ATTR therapy	78 (59%)	64 (55%)	14 (82%)	0.04
Tafamidis	71 (53%)	64 (55%)	7 (41%)	–
Inotersen	2 (2%)	0	2 (12%)	–
Patisiran	5 (4%)	0	5 (29%)	–
Biochemical				
Troponin-T (ng/L)	61 (39, 87)	63 (49, 92)	50 (29, 63)	0.07
NTproBNP (ng/L)	1984 (871, 2537)	2171 (1082, 2579)	1688 (767, 1936)	0.20
eGFR (mL/min/1.73m^2^)	49 (43, 52)	47 (42, 54)	49 (44, 56)	0.83
Echocardiographic				
LV mass index (g/m^2^)	156 (139, 170)	159 (145, 191)	147 (133, 152)	0.13
LV ejection fraction (%)	53 (48, 56)	51 (46, 57)	56 (50, 61)	0.38
LV GLS (%)	13.6 (11.8, 16.7)	13.1 (11.5, 16.0)	14.2 (11.8, 17.2)	0.53

Categorical variables are reported as frequency (percentage) and continuous variables are reported as mean (± standard deviation) or median (interquartile range). *ATTR-CM*-transthyretin amyloidosis cardiomyopathy, *eGFR* Estimated glomerular filtration rate, *GLS* Global longitudinal systolic strain, *hATTR* Hereditary transthyretin amyloidosis, *LV* Left ventricle, *NTproBNP-N* Terminal pro-B-type natriuretic peptide, *NYHA* New York Heart Association, *wtATTR* Wild-type transthyretin amyloidosis

### Evaluation, prevalence and incidence of oeCAD

**Fig. 1:**
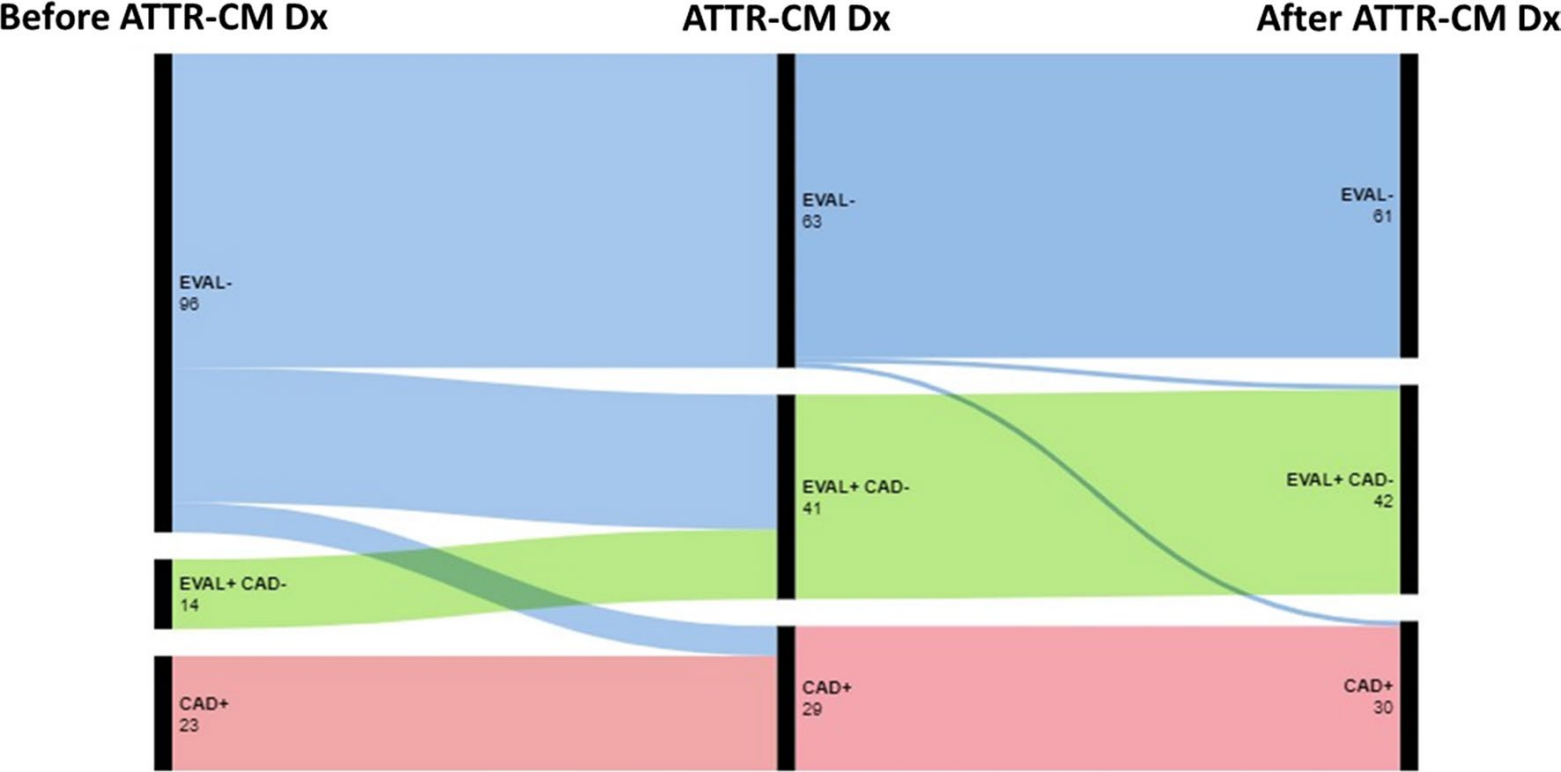
Study flow chart illustrating the time course of obstructive epicardial coronary artery disease (oeCAD) evaluation (EVAL) and diagnosis for transthyretin amyloidosis cardiomyopathy (ATTR-CM) patients, occurring either before, during or after ATTR-CM work-up/diagnosis (Dx). EVAL- indicates no oeCAD evaluation was performed, while EVAL + indicates an oeCAD evaluation was performed. CAD- indicates that a diagnosis of oeCAD was excluded after evaluation, while CAD + indicates an oeCAD diagnosis was confirmed (by invasive coronary angiography in all patients)

Overall, there were 72 (54%) ATTR-CM patients who underwent some form of invasive or non-invasive investigation for the assessment of known or suspected oeCAD, and 61 (46%) who did not undergo any investigation for oeCAD. The indications for oeCAD evaluation are listed in Table 2. Dyspnea and chest pain were the most common indications, followed by patients presenting with ACS and those being investigated for abnormal resting ECG. Among these 72 ATTR-CM patients, 37 (51%) underwent investigations prior to being diagnosed with ATTR-CM (range 21 months to 38 years prior to ATTR-CM diagnosis), of which 23 (62%) were confirmed to have oeCAD. There were 33 (46% of those ever investigated for oeCAD) ATTR-CM patients who underwent investigations as part of their ATTR-CM diagnostic work-up, of which only 6 (18%) were confirmed to have oeCAD. Only 2 (3% of those ever investigated for oeCAD) patients underwent oeCAD investigation following their ATTR-CM diagnosis, including one patient who had syncope and a negative oeCAD work-up, and another presenting with ACS and found to have multi-vessel oeCAD who was subsequently managed medically (further details provided below). Figure 1 presents a flow chart for the study cohort, describing the proportion of patients who underwent an evaluation for oeCAD at varying time points during their ATTR-CM care pathway, and whether the investigations placed them in a positive or negative category for oeCAD.

**Table 2:** Indications for oeCAD evaluation among ATTR-CM patients

Parameter	Total (*N* = 72)
Dyspnea	20 (28%)
Chest pain	19 (26%)
Acute coronary syndrome	18 (25%)
NSTEMI	12 (67%)
STEMI	6 (33%)
Abnormal ECG	6 (8%)
Pre-cardiac surgery / procedure	4 (6%)
Syncope	3 (4%)
Ventricular arrhythmia	2 (3%)

ATTR-CM-transthyretin amyloidosis cardiomyopathy, ECG-electrocardiogram, NSTEMI-non-ST-segment elevation myocardial infarction, oeCAD-obstructive coronary artery disease, STEMI-ST-segment elevation myocardial infarction.

There were 48 ATTR-CM patients (36% of the total study population) whose oeCAD evaluation included invasive coronary angiography, among whom 30 (65%) received a confirmed diagnosis of oeCAD (including 18 patients presenting with ACS), and 18 patients (35%) identified to have no oeCAD. A total of 27 (20% of the total study population) patients had myocardial perfusion imaging performed, including 26 who had single photon emission computed tomography and 1 stress-perfusion cardiac magnetic resonance imaging. Seven (26%) of these patients demonstrated a mild/small perfusion defect, and the remainder demonstrated normal perfusion. Three (11%) of these 7 patients went on to invasive testing and had oeCAD confirmed by coronary angiography in the setting of persistent symptoms, and the remainder had no further investigations for oeCAD. Twelve (44%) of the patients who underwent myocardial perfusion imaging had this test performed as part of the work-up for their clinical presentation with ATTR-CM.

### Characteristics ATTR-CM patients with oeCAD

**Fig. 2:**
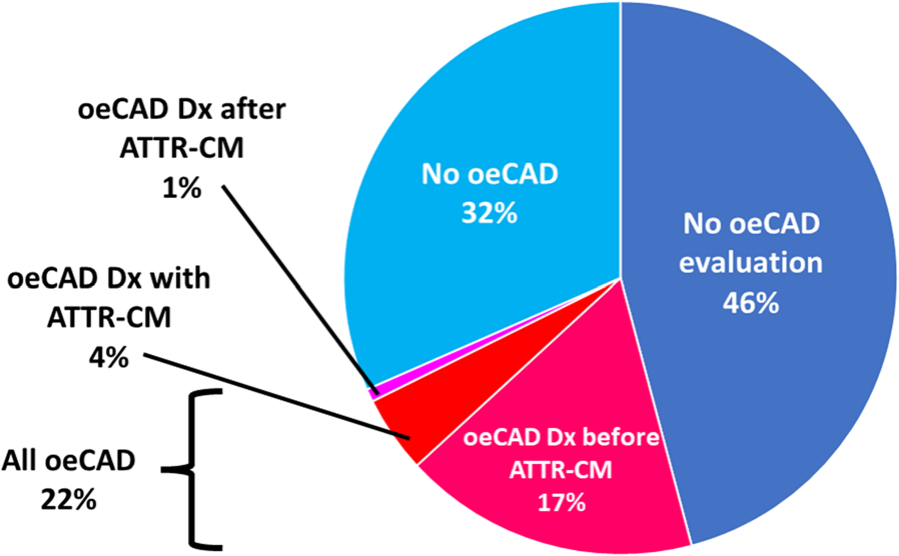
Prevalence of obstructive epicardial coronary artery disease (oeCAD) among transthyretin amyloidosis cardiomyopathy (ATTR-CM) patients, indicating the timing of oeCAD diagnosis as occurring either before, during or after their ATTR-CM work-up/diagnosis

**Fig. 3:**
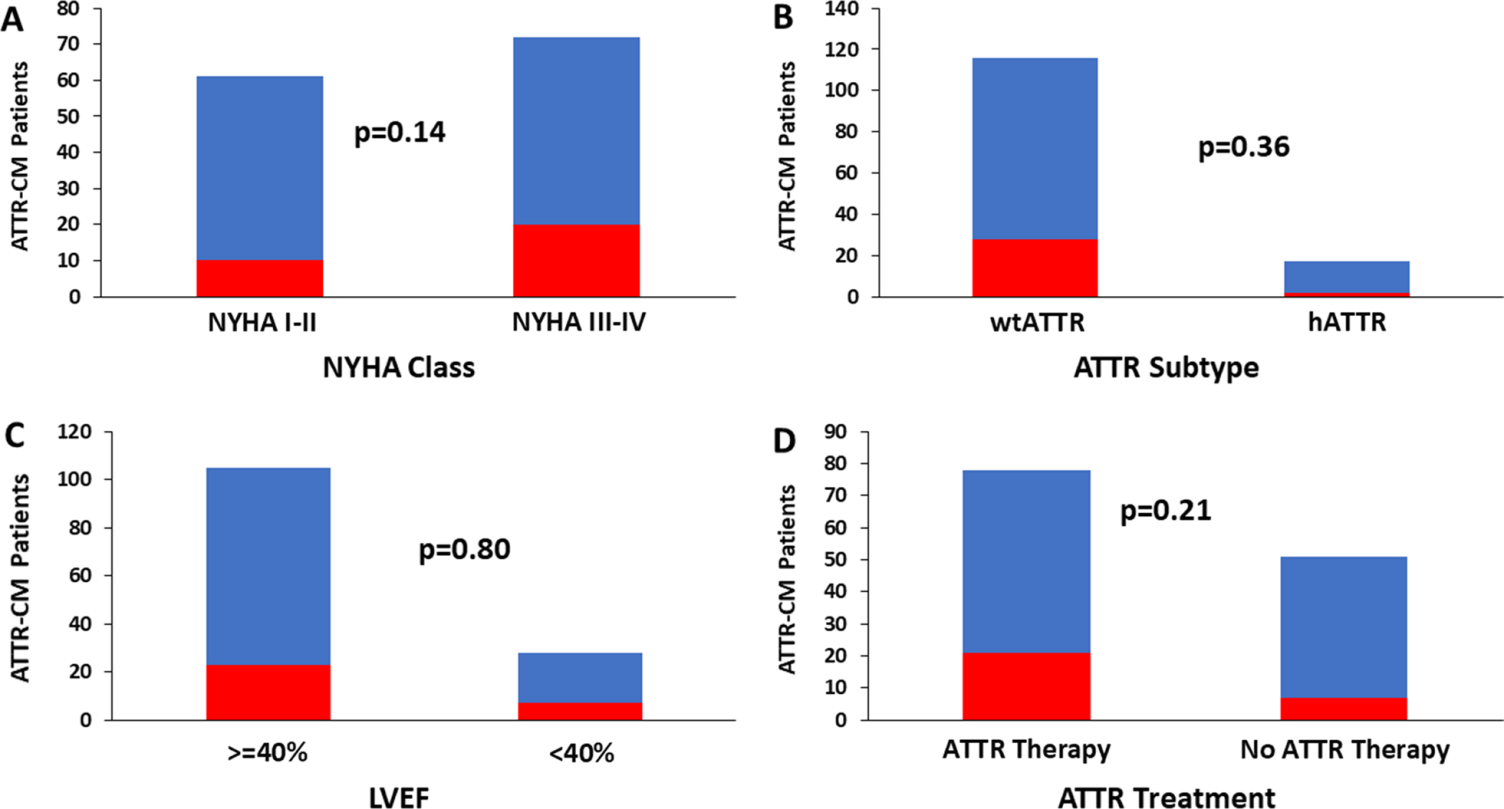
Comparison of the prevalence of obstructive epicardial coronary artery disease (oeCAD) among subgroups of transthyretin amyloidosis cardiomyopathy (ATTR-CM) patients. Red bars indicate ATTR-CM patients with confirmed oeCAD and blue bars indicate the remainder of the cohort. **A** New York Heart Association (NYHA) functional class, **B** ATTR subtype, wild-type (wtATTR) or hereditary (hATTR), **C** left ventricular ejection fraction (LVEF), and **D** use of disease modifying ATTR therapy

Figure 2 reports the prevalence of oeCAD among ATTR-CM patients. There were 30 (42% of those evaluated for oeCAD) ATTR-CM patients with confirmed oeCAD (all confirmed by invasive coronary angiography). Baseline characteristics for ATTR-CM patients with confirmed oeCAD compared with those without oeCAD are shown in Table 3. All clinical, cardiovascular risk factor ATTR specific, medication, biochemical and imaging characteristics were statistically similar between groups. Figure 3 compares the prevalence of oeCAD among select ATTR-CM patient subgroups.

**Table 3:** Comparison of baseline characteristics for ATTR-CM patients with and without oeCAD

Parameter	oeCAD (*n* = 30)	No oeCAD (*n* = 42)	*p*-value
*Clinical Characteristics*			
Age (years)	79 ± 7	78 ± 9	0.72
Male	28 (93%)	38 (90%)	0.99
Body mass index (kg/m^2^)	29 ± 5	27 ± 4	0.79
NYHA functional class III-IV	20 (67%)	21 (50%)	0.23
Atrial fibrillation	14 (47%)	12 (29%)	0.14
Hypertension	12 (40%)	11 (26%)	0.31
Diabetes Mellitus	10 (43%)	7 (17%)	0.16
Hyperlipidemia	8 (27%)	12 (29%)	0.99
Smoking history	6 (20%)	3 (7%)	0.15
*ATTR characteristics*			
wtATTR	28 (93%)	36 (86%)	0.46
hATTR	2 (7%)	6 (14%)	
pV142I	1 (3%)	2 (5%)	0.99
pV50M	0	2 (5%)	0.51
pT80A	1 (3%)	1 (2%)	0.99
other	0	1 (2%)	0.99
*Medications*			
Diuretics	22 (73%)	28 (67%)	0.61
Anticoagulation	16 (53%)	15 (36%)	0.16
ATTR therapy	21 (70%)	23 (55%)	0.23
Tafamidis	21 (70%)	20 (48%)	0.09
Inotersen	0	1 (2%)	0.99
Patisiran	0	2 (5%)	0.51
*Biochemical*			
Troponin-T (ng/L)	71 (46, 85)	54 (32, 65)	0.24
NTproBNP (ng/L)	2218 (1672, 2625)	1842 (767, 2107)	0.16
eGFR (mL/min/1.73m^2^)	47 (41, 50)	51 (43, 56)	0.86
*Echocardiographic*			
LV mass index (g/m^2^)	167 (144, 189)	152 (128, 163)	0.22
LV ejection fraction (%)	48 (45, 54)	56 (50, 59)	0.18
LV GLS (%)	12.9 (10.8, 14.6)	14.1 (12.0, 17.5)	0.64

Categorical variables are reported as frequency (percentage) and continuous variables are reported as mean (± standard deviation) or median (interquartile range). *ATTR-CM* Transthyretin amyloidosis cardiomyopathy, *eGFR* Estimated glomerular filtration rate, *GLS* Global longitudinal systolic strain, *hATTR* Hereditary transthyretin amyloidosis, *LV* Left ventricle, *NTproBNP* N-terminal pro-B-type natriuretic peptide, *NYHA* New York Heart Association, *oeCAD* Obstructive epicardial coronary artery disease, *wtATTR* Wild-type transthyretin amyloidosis

Among patients with confirmed oeCAD, 23 (77%) received this diagnosis before being diagnosed with ATTR-CM, including 17 with prior ACS (11 [65%] with NSTEMI and 6 [35%] with STEMI) and 18 with prior revascularization (11 [61%] with PCI and 7 [39%] with CABG). Of note, among patients with a prior diagnosis of oeCAD, none required further oeCAD-related investigations, interventions or hospitalization after being diagnosed with ATTR-CM. There were 6 (20% of patients with confirmed oeCAD) patients diagnosed with oeCAD during their work-up for ATTR-CM. One patient went on to have CABG and the other 5 patients were managed medically. Again, none of those 6 patients required further investigations, interventions or hospitalization for oeCAD in follow-up. One ATTR-CM patient was diagnosed with oeCAD 13-months after their diagnosis with ATTR-CM, presenting to hospital with NSTEMI and invasive coronary angiography confirming multi-vessel oeCAD. This patient was managed medically and has not required subsequent investigations, interventions or hospitalizations for oeCAD. Among all clinical, cardiovascular risk factor, ATTR-specific, medication, biochemical and imaging variables listed in Table 3, none were significantly associated with a diagnosis of oeCAD among ATTR-CM patients using logistic regression analysis (data not shown). Among wtATTR patients only, 28 (24%) had a diagnosis of oeCAD (*p* = 0.36). Similarly, no characteristics listed in Table 3 were significantly associated with a diagnosis of oeCAD among wtATTR patients only.

### Outcomes of ATTR-CM patients with and without oeCAD

At a median follow-up of 27 months (interquartile range 18, 35 months) 37 (28% of the total study population) ATTR-CM patients died, including 5 (17%) deaths of patients with oeCAD and 32 (31%) deaths of ATTR-CM patients in the remainder of the cohort (including 13 in patients with no oeCAD and 19 in patients having no oeCAD investigation). Using Cox proportional hazards modeling, the presence of oeCAD was not significantly associated with mortality (HR 0.72, 95% CI 0.28–1.94, *p* = 0.53). Cardiovascular risk factors were not significantly associated with mortality among ATTR-CM patients. Among wtATTR patients only, 36 (31%) patients died, including 5 patients with oeCAD. The presence of oeCAD was not significantly associated with mortality among wtATTR patients only.

There were 56 (42% of the total study population) ATTR-CM patients hospitalized during the follow-up period, including 10 (18%) patients with oeCAD (6 cardiovascular hospitalizations and 4 non-cardiovascular hospitalizations), and 46 (82%) in the remainder of the cohort (29 cardiovascular hospitalizations and 17 non-cardiovascular hospitalizations). There were 13 hospitalizations in the follow-up period among patients without oeCAD (including 8 cardiovascular and 5 non-cardiovascular) and 33 in patients have no oeCAD investigation (including 21 cardiovascular and 12 non-cardiovascular). Using Cox proportional hazards modeling, the presence of oeCAD was not significantly associated with the composite outcome of death or hospitalization (HR 0.85, 95% CI 0.41–1.79, *p* = 0.67). Cardiovascular risk factors were not significantly associated with the composite outcome of death or hospitalization among ATTR-CM patients. Among wtATTR patients only, 51 (44%) patients were hospitalized, including 9 patients with oeCAD (including 6 cardiovascular hospitalizations and 3 non-cardiovascular hospitalizations). The presence of oeCAD was not significantly associated with the composite outcome of death or hospitalization among wtATTR patients only.

## Discussion

This study is the first to describe the prevalence of oeCAD among patients with ATTR-CM and its associations to major clinical outcomes. The main findings are as follows: (1) approximately one quarter of ATTR-CM patients in our cohort had concurrent oeCAD, the majority of whom were diagnosed prior to ATTR-CM diagnosis, (2) upon clinical presentation with ATTR-CM, most patients underwent some form of oeCAD-related investigation, the majority of these being negative, (3) among ATTR-CM patients with oeCAD, the majority did not exhibit manifestations of this at the time of ATTR-CM diagnosis and during follow-up., and (4) a diagnosis of oeCAD was not associated with adverse outcomes in ATTR-CM patients. Overall, these findings suggest that, while oeCAD is prevalent among patients with ATTR-CM, it typically precedes ATTR-CM diagnosis. While an association between a diagnosis of oeCAD and adverse outcomes in ATTR-CM patients was not found in this analysis, this cannot be excluded due to the study methodology and sample size limitations of this cohort.

Cardiac amyloidosis is recognized to be associated with intramural microvascular disease in the absence of oeCAD, and much of this evidence comes from histopathologic studies of AL amyloidosis patients [3]. This small vessel disease can result in symptoms of angina, and may contribute to unstable coronary syndromes or progressive HF [7]. Multiple reports have described cardiac amyloidosis presenting with signs and/or symptoms of ischemic heart disease [12, 13]. It has been reported that between 10–20% of cardiac amyloidosis patients initially present with symptoms of chest pain, and that up to two-thirds of patients have significant intramural coronary amyloid deposits, many associated with focal microscopic changes of ischemic injury [14, 15]. Older pathologic studies describe the prevalence of intramural coronary artery amyloid deposits as much lower in ATTR-CM compared with AL cardiac amyloidosis 16–18], while more recent studies have confirmed coronary amyloid deposits in ATTR-CM patients [6]. Perivascular and interstitial myocardial deposits may cause compression of coronary microvasculature that can also contribute to symptoms of ischemic heart disease [19]. Significant coronary microvascular dysfunction was demonstrated by Dorbala, et al. in a mixed cardiac amyloidosis subtype (ATTR and AL) cohort of patients without epicardial coronary artery disease undergoing N-13 ammonia positron emission tomography, and patients had reduced peak vasodilator stress myocardial blood flow and flow reserve [19].

While amyloid infiltration of the epicardial coronary artery wall is also common in histopathologic studies, it generally does not result in obstructive disease [14]. Our study is consistent with earlier reports that a clinical diagnosis of oeCAD is rare once patients receive a diagnosis of ATTR-CM [4, 6], and that most patients with oeCAD are diagnosed prior to receiving a ATTR-CM diagnosis. Because of similarities in clinical presentation between oeCAD and cardiac amyloidosis, many ATTR-CM patients undergo investigation for oeCAD at the time of presentation. Beyond similar signs and symptoms, many cardiac amyloidosis patients have ECG abnormalities that may mimic oeCAD, and many have chronically elevated troponin values. In our study, the majority of investigations for oeCAD at the time of ATTR-CM diagnosis were negative. It has been postulated that cardiac amyloidosis may accelerate pre-existing atherosclerotic epicardial coronary artery diease via the deposition of amyloid deposits around coronary vessels [7, 8], however the findings from our study do not support this in patients with ATTR-CM. Mortality and hospitalization rate were similar between patients with and without oeCAD, and very few patients required further investigations for oeCAD after ATTR-CM diagnosis. Although beyond the scope of this study to confirm, these findings might suggest that medications commonly used to treat oeCAD that are poorly tolerated in patients with ATTR-CM, especially beta-blockers, may be safely reduced or even discontinued in patients with a prior oeCAD diagnosis, although this requires further research, and a cautious approach to titration of these medications in this setting is still recommended.

### Limitations

There are a number of important limitations to our analysis. This was a single-center, retrospective, observational cohort study, and therefore the presence of bias cannot be excluded. Importantly, not all patients included in this observational cohort study underwent evaluation for oeCAD, and so the presence of oeCAD in these patients cannot be excluded. Notably, there were 4 patients who had a small/mild perfusion defect on myocardial perfusion imaging who were not subsequently referred for coronary angiography, and so the presence of oeCAD cannot be excluded in these patients. Patients did not undergo evaluation for microvascular dysfunction, and therefore the prevalence of this and its impact on our results is uncertain. Similarly, autopsy data was not available for deceased patients, and so correlation of clinical with histopathologic findings cannot be performed, although several previous studies have examined this. Our report lacks detailed descriptions of medical management changes for ATTR-CM patients, including anti-anginal agents often used to treat oeCAD. A recent report describing rates of beta-blocker prescription among a mixed subtype cohort of cardiac amyloidosis patients found that their use was not infrequent, with approximately 1/3 of patients continuing to take them after diagnosis, the majority for treatment of arrhythmia [20].

## Conclusions

oeCAD is prevalent among ATTR-CM patients, however is typically known at time of ATTR-CM diagnosis. Characteristics of ATTR-CM patients with and without oeCAD are similar. ATTR-CM patients with oeCAD typically did not require additional investigations or oeCAD-related interventions during follow-up. While an association between oeCAD and adverse outcomes in ATTR-CM patients was not identified, this cannot be excluded due to methodologic limitations of this analysis and confirmation is needed by larger prospective studies.

## Data Availability

The datasets used and/or analyzed during the current study are available from the corresponding author on reasonable request.

## Abbreviations

ACS: Acute coronary syndrome
ATTR: CM: transthyretin amyloidosis cardiomyopathy
CABG: Coronary artery bypass grafting
ECG: Electrocardiogram
hATTR: Hereditary transthyretin amyloidosis
MI: Myocardial infarction
oeCAD: Obstructive epicardial coronary artery disease
PCI: Percutaneous coronary intervention
TTR: Transthyretin protein
wtATTR: Wild: type transthyretin
